# CO Spillover Is Not a Promoter for C─C Bond Formation in CO_2_ Electroreduction on Cu‐Ag Bimetallic Catalysts

**DOI:** 10.1002/advs.202520469

**Published:** 2026-03-02

**Authors:** Beining Xu, Zhaochun Liu, Xinjuan Du, Wen Yan, Yunsong Yu, Ionut Tranca, Frederik Tielens, Ming Ma

**Affiliations:** ^1^ School of Chemical Engineering and Technology Xi'an Jiaotong University Xi'an P. R. China; ^2^ General Chemistry (ALGC) Materials Modelling Group Vrije Universiteit Brussel (VUB) Brussels Belgium

**Keywords:** CO spillover, CO_2_ electroreduction, Cu‐based bimetallic catalysts, spillover effect

## Abstract

Cu‐based bimetallic catalysts have been shown to improve the multi‐carbon (C_2+_) selectivity in CO_2_ electroreduction, with the assumption that CO spillover from the CO‐selective catalysts to adjacent Cu domains via their bimetallic interface promotes C–C coupling. Here, through systematically controlling the Cu‐Ag interface densities of bimetallic catalysts, we report that CO spillover via the bimetallic interface is unlikely to enhance the formation of C_2+_ products. Conversely, the abundant Cu‐Ag interface preferentially promotes CH_4_ formation while suppressing C─C bond formation. CO stripping studies also reveal that the Cu‐Ag interface does not favor C─C coupling. Further computational modelling suggests that Cu‐Ag bimetallic interface significantly enhances the energy barrier of CO dimerization while lowering the energy barrier for ^*^CO hydrogenation toward CH_4_, thus inhibiting C─C coupling toward C_2+_ products while facilitating CH_4_ formation.

## Graphical abstract

CO spillover via the Cu‐Ag interface does not promote C‐C coupling.

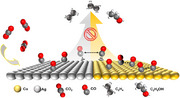

## 1 Introduction

The electrochemical reduction of CO_2_ into high‐value chemicals represents one of the most promising avenues for maintaining balance in the carbon cycle and developing a sustainable society [1, 2, 3]. In CO_2_ electrolysis field, the synthesis of multi‐carbon hydrocarbons and oxygenates (C_2+_), such as ethylene (C_2_H_4_) and ethanol (C_2_H_5_OH), has attracted considerable attention due to their widespread market potential and high energy density [4, 5, 6, 7, 8]. Cu has been identified to have the capability of electrochemically converting CO_2_ into various C_2+_ products with appreciable selectivity at high reaction rates under mild conditions, but steering the selectivity toward the synthesis of a specific C_2+_ product on the Cu surface remains a critical challenge [9, 10, 11, 12, 13].

The electrosynthesis of C_2+_ products is a complex reaction that involves the formation of numerous intermediates with multiple electron transfer steps, the formation and coverage of the key intermediates that are related to C─C coupling play an essential role in the reaction pathways toward a desired C_2+_ product in CO_2_ electrolysis [13, 14, 15, 16]. In the most accepted theory, CO is an essential intermediate for C─C coupling, and it was also demonstrated that high CO coverage favors C─C coupling toward C_2+_ products [17, 18, 19, 20, 21, 22, 23, 24]. In this context, Cu‐based tandem catalysis, in which a CO‐selective catalyst (such as Au, Ag, Zn) provides the source of CO for nearby Cu active sites, has gained significant interest as an appealing strategy for improving the catalytic performance toward the formation of specific C_2+_ products.

As a typical tandem catalysis in the CO_2_ electrolysis field, Cu‐based bimetallic catalysts have been widely explored to enhance C_2+_ products. For instance, the Buonsanti group reported that Cu‐Ag nanodimer catalysts could facilitate C‐C coupling via their interface, leading to a 3.4‐fold enhancement in Faradaic efficiency (FE) toward C_2_H_4_ in comparison with the pure Cu counterpart [17]. Additionally, a Cu‐Au bimetallic catalyst prepared by depositing nanoparticles on a flat polycrystalline Cu foil showed a highly enhanced catalytic activity for the reduction of CO_2_ to C_2+_ alcohols compared to a single Cu foil [25]. Furthermore, Ren et al. synthesized oxide‐derived Cu‐Zn catalysts with tunable amounts of Zn, demonstrating a Faradaic efficiency of up to 29.1% for ethanol on the Cu_4_‐Zn bimetallic catalyst [26]. All of the above studies indicate that Cu‐based bimetallic catalysts are capable of promoting the formation of C_2+_ products. To explain the improved performance toward a specific C_2+_ product on these bimetallic catalysts, a spillover mechanism has been proposed [17, 25, 26, 27, 28, 29, 30, 31], where one CO‐selective catalyst generates CO and subsequently migrates to the adjacent Cu domains for C─C coupling. Although this CO spillover has been widely used for explaining variable C_2+_ selectivity on the Cu‐based tandem catalysis in the CO_2_ electrolysis field, direct evidence that CO spillover at the interfacial boundary of two different metals on Cu‐based binary catalysts enhances specific C_2+_ products is lacking to date. Additionally, the majority of Cu‐based bimetallic catalysts in previous work were based on nanostructured morphology (as summarized in Table S3), synthesized via chemical methods. In comparison with the Cu counterpart, the process for introducing CO‐selective metal catalysts into Cu may inadvertently create complex reactive sites, along with potential variation in the nanostructure. Thereby, this commonly held CO spillover assumption deserves scrutiny.

Herein, to circumvent the potential variation in morphology and the possible creation of complex active sites during the fabrication of bimetallic catalysts, Cu‐Ag bimetallic catalysts with controllable interfacial densities were synthesized via physical vapor deposition. Via comparing CO_2_ reduction reaction (CO_2_RR) and CO reduction reaction (CORR) on variant bimetallic interface densities, we demonstrate that the catalytic selectivity of total C_2+_ products is gradually inhibited with enhanced methane (CH_4_) formation upon increasing the Cu‐Ag interface densities. This finding indicates that CO spillover via the bimetallic interface is minimal in promoting the C─C bond formation on bimetallic catalysts. Furthermore, computational calculations imply that Cu‐Ag interfaces promote the direct CO hydrogenation instead of CO dimerization, correspondingly leading to the facile formation of CH_4_ with suppressed production of C_2+_ during CO_2_/CO reduction.

## 2 Results and Discussion

### 2.1 Preparation and Characterization of Cu‐Ag Bimetallic Catalysts

**FIGURE 1:**
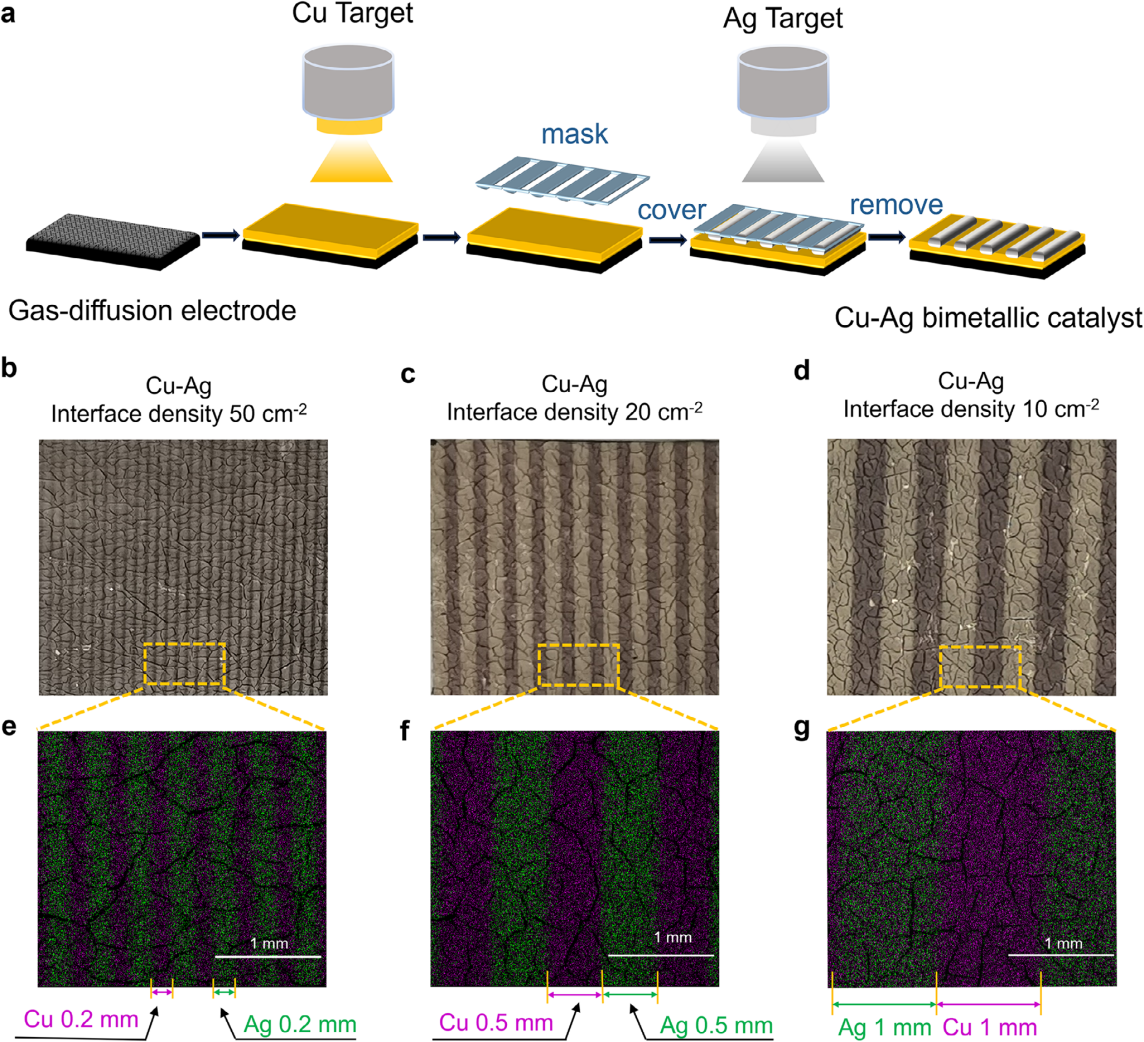
Preparation and characterization of Cu‐Ag bimetallic catalysts. (a) Schematic illustration of the preparation process of Cu‐Ag bimetallic catalysts with controllable interface densities via deposition masks. The digital photos and the related EDS elemental mapping images (Cu, purple; Ag, green) for the typical Cu‐Ag binary samples with interface densities of (b, e) 50 cm^−2^, (c, f) 20 cm^−2^ and (d, g) 10 cm^−2^, respectively (The Cu and Ag catalysts were arranged alternately at equal intervals with 0.2, 0.5, and 1 mm, respectively).

Using direct current (DC) magnetron sputtering (50 W), ∼45 nm thick Cu catalyst layers were initially deposited on gas diffusion electrodes, as shown in Figure 1a. To obtain Cu‐Ag bimetallic catalysts with explicit interface boundaries, the Cu layers were covered by deposition masks featuring slits, and subsequently Ag electrocatalysts with ∼45 nm thick layers were prepared on the mask‐covered Cu layer via DC magnetron sputtering (Figure 1a). Here, the deposition masks with tailored slit widths of 0.1, 0.2, 0.5, 1 and 5 mm were employed separately (Figure S1), thus enabling us to synthesize Cu‐Ag binary catalysts with controllable interface densities. Notably, slit width always equals shield width for all different masks (Figure S2), which provides the identical total surface area of Cu for all the binary catalysts (i.e., the total surface area of Cu is equal to that of Ag), thus circumventing the effect of Cu surface area in different samples in this work.

The typical digital images of the binary samples imply an obvious increase in interface density of the two metals upon decreasing the slits widths of masks (Figure 1b–d). For simplification, the number of bimetallic interfaces per unit area (cm^2^) is defined as the interface density in this work. For instance, an interface density of 50 cm^−2^ was prepared via the deposition masks with slit widths of 0.2 mm. To further identify the distribution of binary metals and their interface densities, energy‐dispersive X‐ray spectroscopy (EDS) was conducted. Figure 1e–g show the typical elemental mapping of Cu‐Ag bimetallic catalysts prepared using deposition masks with different slit widths, where clear Cu‐Ag interfaces were observed for all binary samples. Additionally, via the deposition masks with controllable slit widths, we prepared the Cu and Ag catalysts in an equidistant alternating pattern with strip widths of 0.2, 0.5, and 1 mm (Figure 1e–g), which corresponds to the Cu‐Ag interface densities of 50, 20 and 10 cm^−2^, respectively. Thus, Cu‐Ag binary catalysts with enhanced interface densities were synthesized by reducing the slit widths of masks (Table S1).

### 2.2 Electrochemical Measurements

The electrochemical CO_2_/CORR were conducted in a three‐compartment flow cell, where the catholyte was separated from the anolyte by an anion exchange membrane. The feed gas, comprising either pure CO_2_ or CO, was continuously supplied into a gas chamber at a constant flow rate of 25 mL/min. During the electrolysis, the gas products mixed with unreacted CO_2_ or CO were directly vented into the gas‐sampling loop of an online gas chromatograph for periodic quantification. The liquid products dissolved in the given catholyte and anolyte reservoirs were collected and subsequently analyzed using high‐performance liquid chromatography. To get a more reliable quantification of liquid products, the influence of water crossover via the membrane on the electrolyte volume was also taken into account in this study [32, 33].

### 2.3 Cu‐Ag Interface Effect on CO_2_RR Performance

**FIGURE 2:**
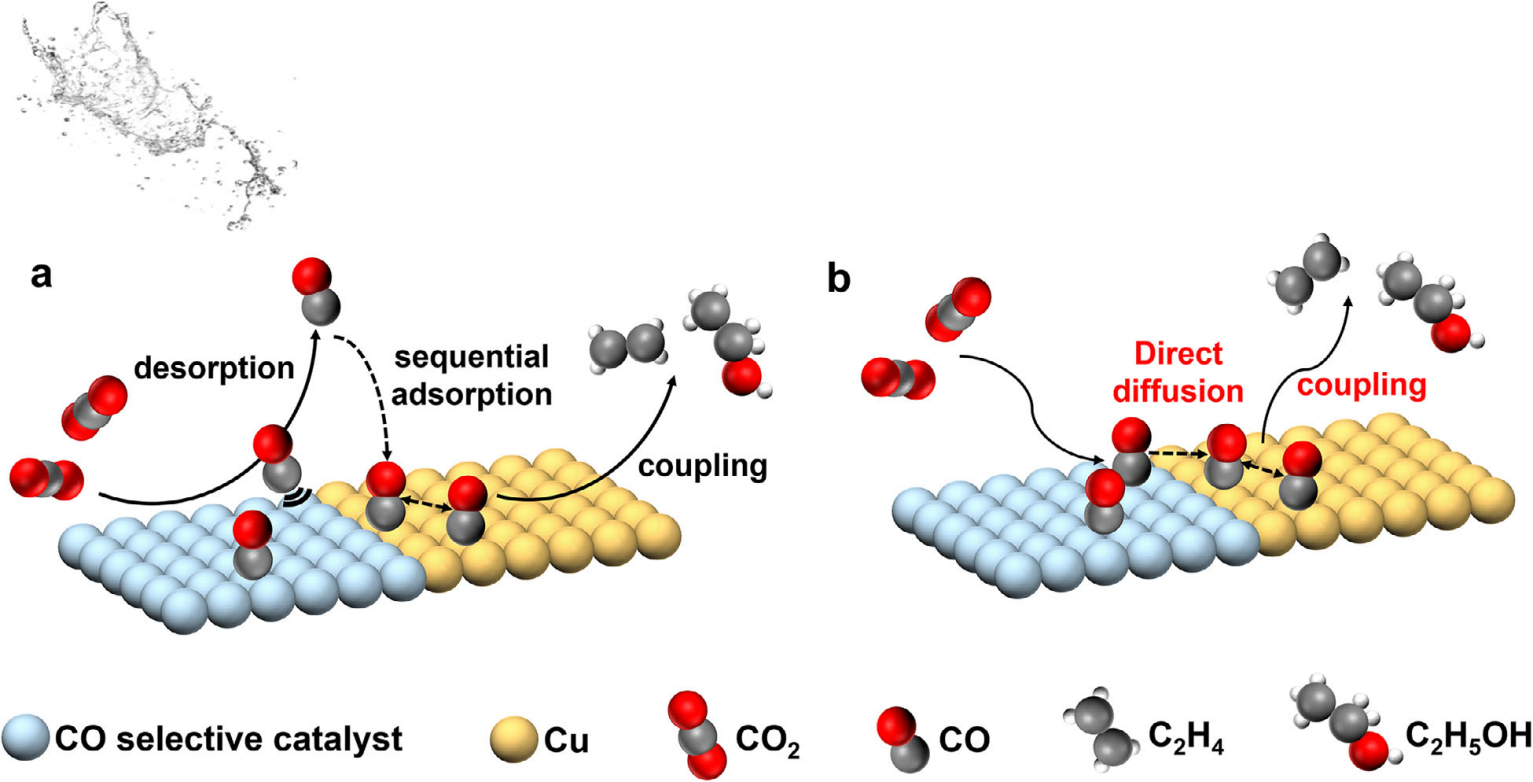
Schematic illustration of the CO spillover mechanism for C_2+_ products promotion on Cu‐based bimetallic catalysts via two possible routes: (a) CO desorption and sequential adsorption, or (b) CO direct diffusion via the interface.

The previous work has shown that Cu‐based bimetallic catalysts consisting of one CO‐selective metal could improve the formation of total C_2+_ products, particularly for the enhancement in the catalytic selectivity and activity for ethanol [22, 34, 35, 36] and C_2_H_4_ [37, 38]. In an attempt to interpret the improved production of ethanol and C_2_H_4_, a CO spillover hypothesis has been proposed and widely accepted as a key reaction procedure on a Cu‐based bimetallic catalyst. For the CO spillover hypothesis, there are two possible routes: (i) CO generated on CO‐selective catalysts desorbs and diffuses to the nearby Cu sites (Figure 2a), and (ii) CO adsorbed on Ag directly diffuses to the Cu sites via the interfacial boundaries between CO‐selective catalysts and Cu (Figure 2b). Regardless of the pattern of routes, CO spillover occurs near the interfacial boundary (Figure 2), which suggests that interface density plays an important role in the specific C_2+_ formation on Cu‐based bimetallic catalysts. Specifically, the interface density should be proportional to C_2+_ formation (such as C_2_H_5_OH or C_2_H_4_). Thus, exploring the correlation between the interface density of two different metals and C_2+_ product formation in CO_2_RR can provide an effective and straightforward approach for better understanding and even verifying the spillover effect.

In this work, we prepared Cu‐Ag bimetallic catalysts using magnetron sputtering, which allows us to obtain controllable interface densities via deposition masks (details in Section 2.1). This deposition technique not only circumvents the variation of morphology, thickness and crystal facets but also offers the identical total surface area of each metal for all the binary catalysts when varying the interface densities, thus enabling us to more accurately correlate the interface density with the formation of C_2+_ products.

**FIGURE 3:**
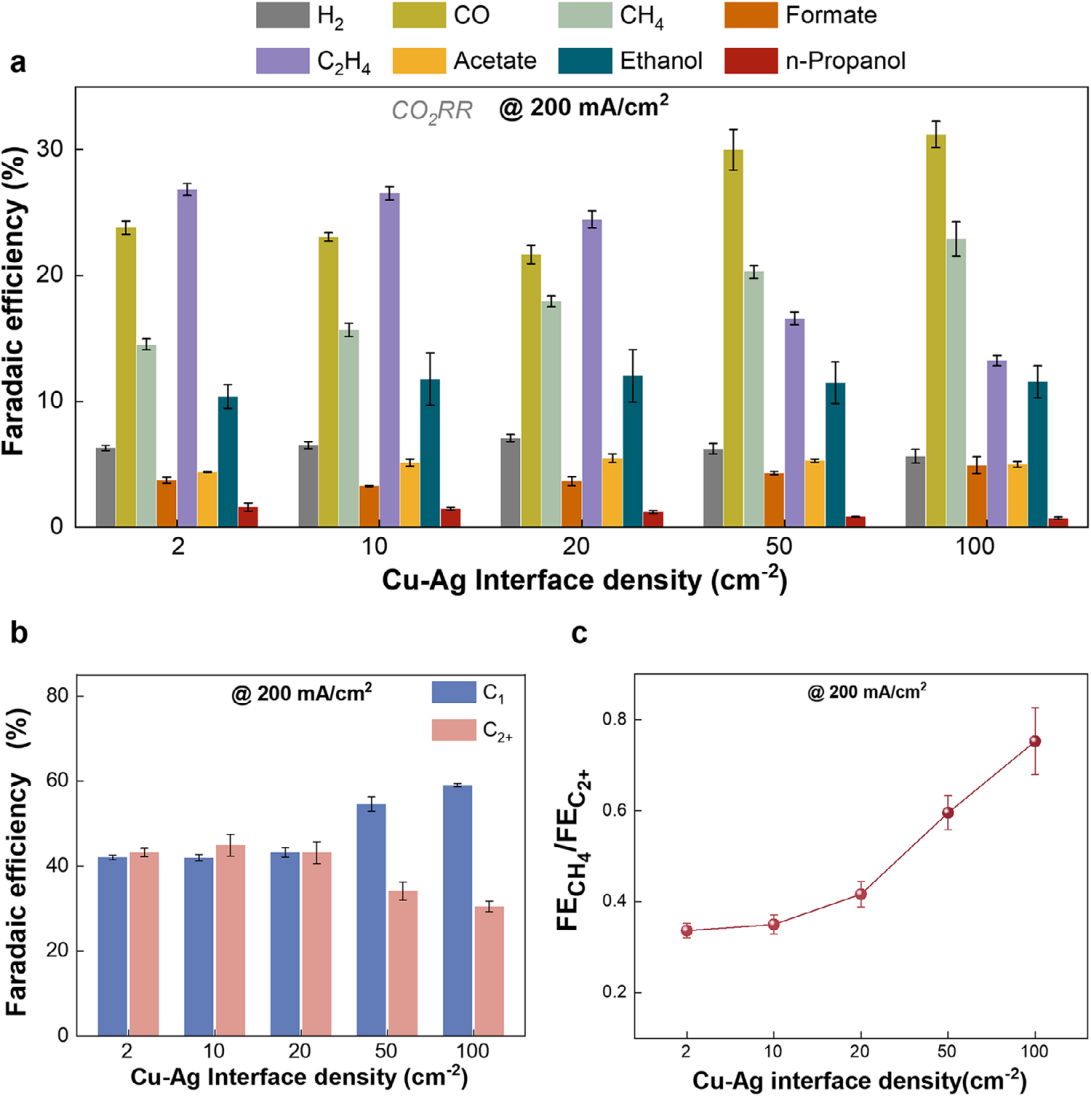
CO_2_ electroreduction performance on Cu‐Ag bimetallic catalysts with different interface density in a flow cell with 1 M KHCO_3_ at 200 mA/cm^2^. (a) Faradaic efficiency for CO_2_ reduction to all detected gas and liquid products. (b) Faradaic efficiency of the C_1_ (including CO, CH_4_, and Formate) and C_2+_ products. (c) Comparative FE of CH_4_ to C_2+_ ratio.

A comparison of the CO_2_RR performance of Cu‐Ag bimetallic catalysts with different interface densities is presented in Figure 3a. We found that hydrogen Faradaic efficiency remained relatively constant (∼7%) when systematically varying the interface densities of the Cu‐Ag bimetallic catalysts. This finding may be linked to the fact that the total surface area of the Ag component is always equal to that of Cu for all the Cu‐Ag binary catalysts when tuning interface densities. Additionally, FE for CH_4_ gradually enhanced upon increasing the interface densities, which may be attributed to that the active sites at the Cu‐Ag interface favor CH_4_ formation (more discussion can be found in the following section). Notably, a decreased FE for total C_2+_ products was observed at a higher interface density (Figure 3b). Instead of promoting C_2+_ formation via the interface boundary, we found an enhancement in the ratio of CH_4_ to C_2+_ with increasing the Cu‐Ag interface density (Figure 3c). These results indicate that C─C coupling that leads to the formation of C_2+_ products is inhibited with a higher Cu‐Ag interface density, which contradicts the generally accepted CO spillover hypothesis. Thus, it seems that the improved C_2+_ formation on Cu‐based bimetallic catalysts in the previous work may not be linked to the CO spillover effect at the interfacial boundary.

Regarding the distribution of specific C_2+_ products, as the interface density of the Cu‐Ag bimetallic catalysts increased, a gradual decline in the FE for C_2_H_4_ was observed, which nearly follows the catalytic trend of the total C_2+_ formation. Of particular note, we found that both ethanol Faradaic efficiency and acetate Faradaic efficiency were almost maintained when varying the interface densities. This finding suggests that the key reaction path toward ethanol and acetate may be separated from that for producing C_2_H_4_, which is consistent with our previous work [24]. More importantly, these results imply that the bimetallic interface is unlikely to improve any specific C_2+_ product via the CO spillover.

### 2.4 Cu‐Ag Interface Effect on CORR Performance

To obtain insights into the role of the CO spillover at the Cu‐Ag interface in the formation of C_2+_ products, CO electrolysis offers a significant approach owing to the following several reasons. Initially, it is well‐known that CO is the key intermediate in the formation of the C─C bond that results in C_2+_ products. Additionally, based on the CO spillover hypothesis, CO formed from Ag should spill over to the adjacent Cu domains for CO─CO coupling. This CO spillover was thought to be one of the main driving forces for the enhanced C_2+_ products in CO_2_ reduction on the bimetallic surface. Thus, when supplying Cu domains with sufficient CO, the CO spillover effect can be negligible for C_2+_ formation on the Cu‐Ag metallic surface.

**FIGURE 4:**
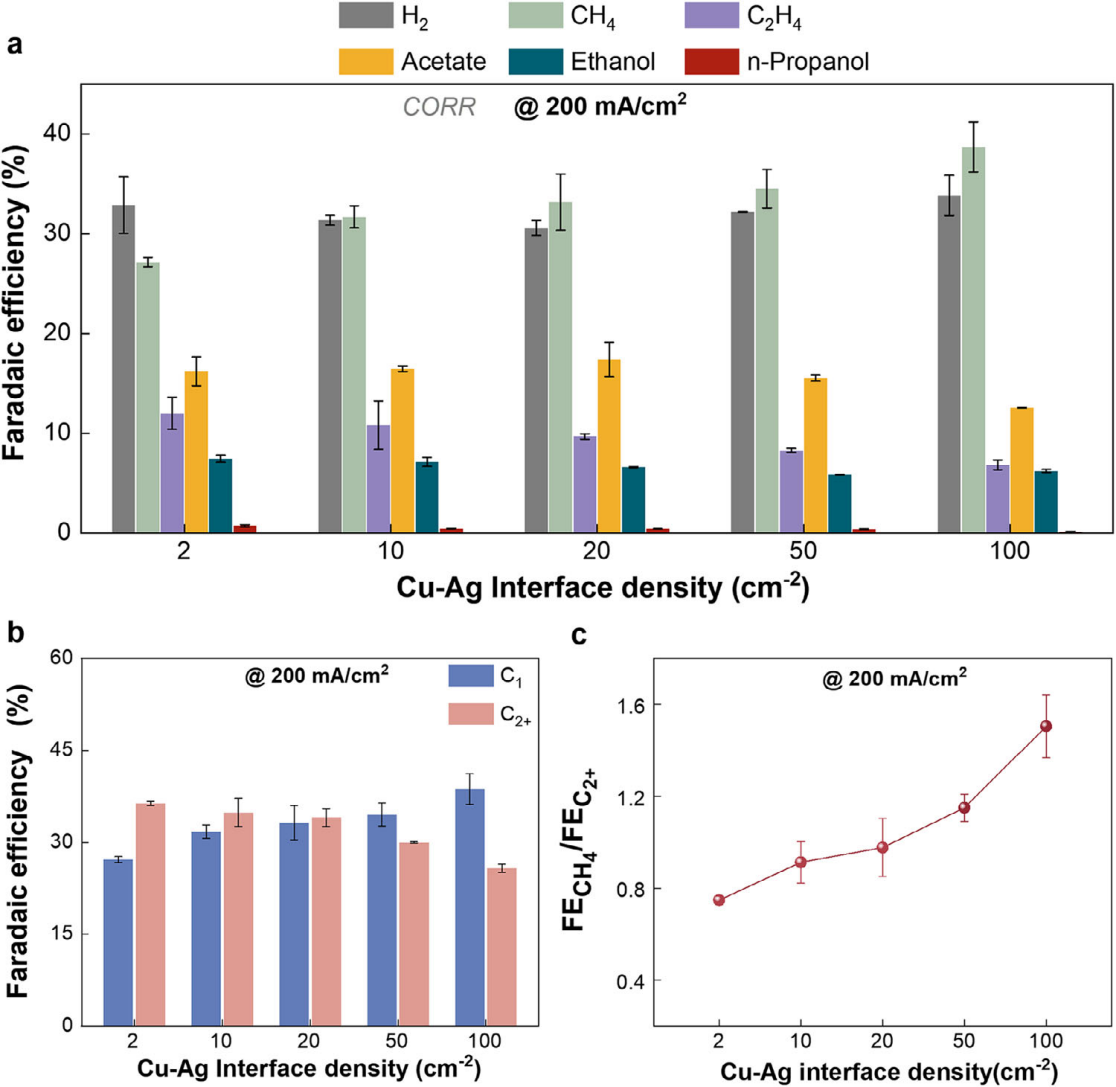
CO electroreduction performance on Cu‐Ag bimetallic catalysts with different interface density in a flow cell with 1 M KHCO_3_ at 200 mA/cm^2^. (a) Faradaic efficiency for CO reduction to all detected gas and liquid products. (b) Faradaic efficiency of the C_1_ (including CH_4_) and C_2+_ products. (c) Comparison of the ratios of FE_CH4_ to FE_C2+_.

In this work, CO electrolysis on Cu‐Ag metallic catalysts with systemically variant interface densities was performed in Gas Diffusion Electrode (GDE)‐type flow electrolyzers that can accelerate CO mass transport (i.e., sufficient CO supply). Figure 4 shows a comparison of the CORR performance of Cu‐Ag bimetallic catalysts with various interface densities. We found more hydrogen formation (more than 31% FE) on the bimetallic surface when feeding CO (Figure 4a) in comparison with that under the feed of pure CO_2_ (Figure 3a), owing to that the Ag surface cannot be involved in CORR but only in H_2_ evolution reaction (Figure S5a). Additionally, more favorable acetate formation (∼16% FE) was detected in CORR than in CO_2_RR. It has been demonstrated that acetate formation is a solution reaction directly affected by OH^−^ concentration in the vicinity of the cathodic GDE surface (i.e., high local pH favors acetate formation) [24, 39]. Thus, the enhanced acetate formation in CORR should be linked to the elevated local pH in the absence of the neutralization reaction between CO_2_ and current‐induced OH^−^ near the cathodic GDE surface. While CORR facilities acetate formation, we found that the selectivity of total C_2+_ products in CORR was lower in comparison with that in CO_2_RR.

Notably, as Cu‐Ag interface densities increased, the formation of C_1_ gradually enhanced while inhibiting the formation of the total C_2+_ products in CORR (Figure 4b). Correspondingly, the ratio of CH_4_ to C_2+_ apparently increased under a higher Cu‐Ag interface density, as shown in Figure 4c. These observations reveal that the catalytic selectivity trend of total C_2+_ products as a function of interface density under sufficient CO supply for Cu domains (avoiding CO spillover effect) is consistent with that in CO_2_RR. In other words, CO spillover through the interfacial boundary between CO‐selective catalysts and Cu should not obviously contribute to the C‐C coupling that leads to C_2+_ products.

**FIGURE 5:**
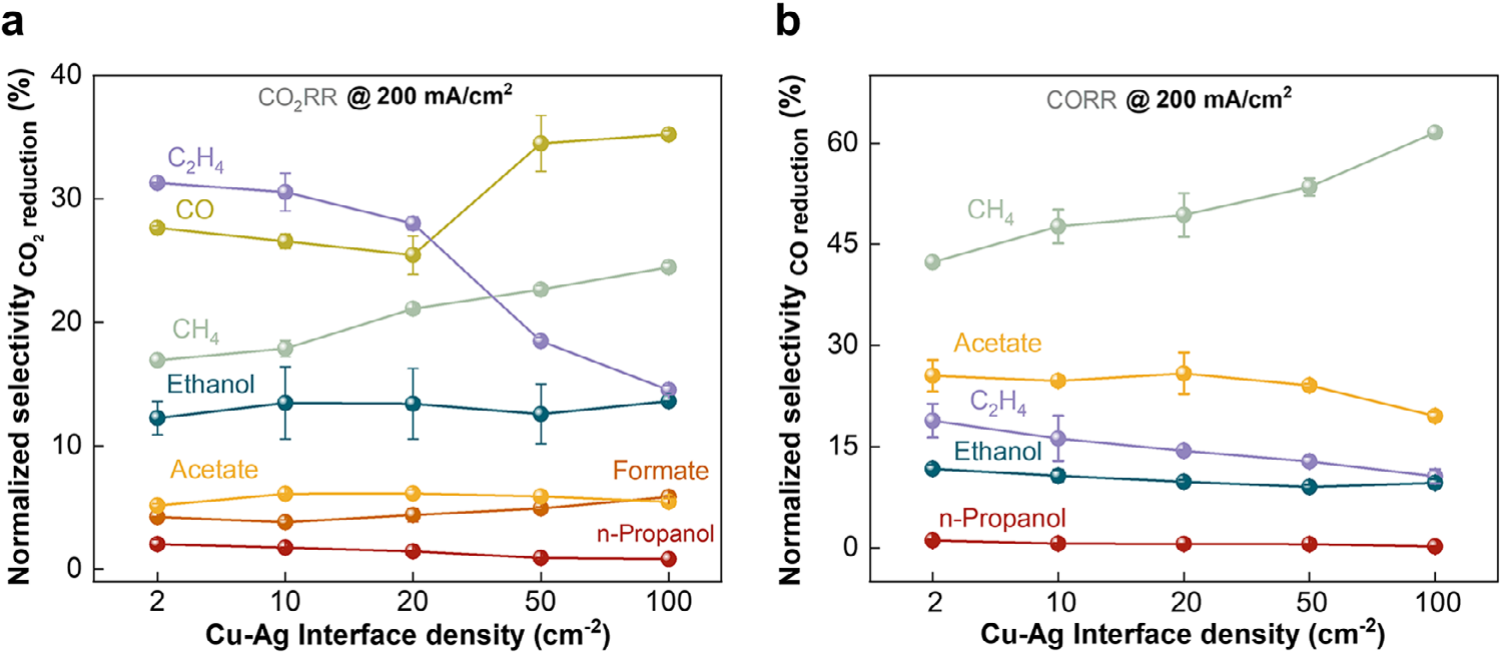
A comparison of CO_2_RR and CORR performance on the Cu‐Ag bimetallic catalysts with different interface density in a flow cell with 1 M KHCO_3_ at 200 mA/cm^2^. (a) Normalized selectivity for CO_2_ reduction products (i.e., excluding H_2_ evolution) via the comparison of FE for certain C_2+_ products with the total FEs for all CO_2_ reduction products. (b) Normalized selectivity for CO reduction products.

To further compare the influence of interface density on specific products, the FEs for products in the CO_2_ reduction and CO reduction were normalized (i.e., excluding the Faraday efficiency of H_2_), respectively. The normalized selectivity for ethanol and acetate only altered slightly with varying interface densities in both CO_2_RR and CORR (Figure 5a,b). In contrast, the normalized selectivity for C_2_H_4_ significantly declined with an increase in the interfacial density of the Cu‐Ag bimetallic catalysts. This observation may be linked to that the key reaction path toward C_2_H_4_ is separated from those for ethanol and acetate formation. Notably, the Cu‐Ag bimetallic interface was unable to facilitate any specific C_2+_ product activity, but promoted the formation of CH_4_.

**FIGURE 6:**
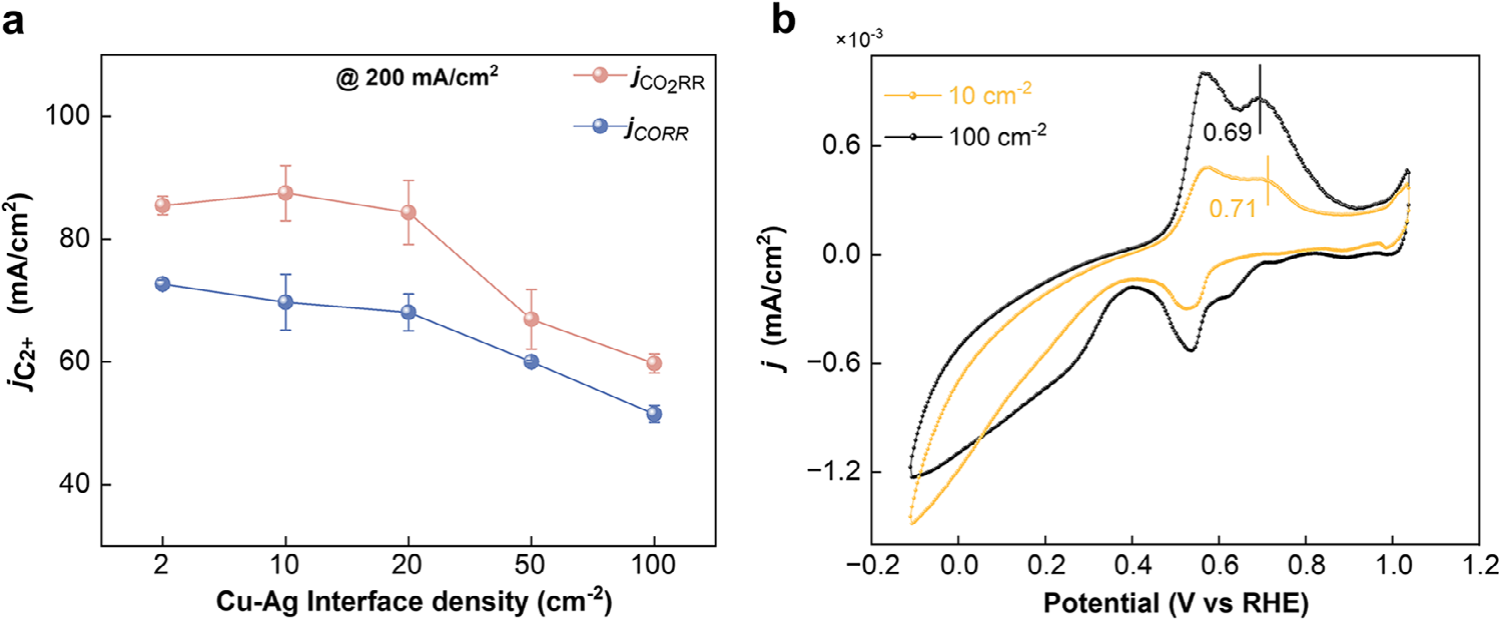
Electrochemical performance of Cu‐Ag bimetallic catalysts with different interface density. (a) A comparison of the total C_2+_ products electrochemical activity of CO_2_RR and CORR on the Cu‐Ag bimetallic catalysts with different interface density at 200 mA/cm^2^. (b) CO stripping curves of Cu‐Ag bimetallic catalysts with a typical interface density of 10 and 100 cm^−2^.

Additionally, a comparison of total C_2+_ formation activity (*j*_C2+_) between CO_2_RR and CORR with different interface density exhibits a nearly identical downward tendency for *j*_C2+_ between CO_2_RR and CORR when increasing the Cu‐Ag interface density (Figure 6a). Based on all the above results, the catalytic trend toward total C_2+_ products in the case of circumventing the CO spillover effect is in line with that in CO_2_RR in the previous section, thereby it is unlikely to have an obvious CO spillover for facilitating C_2+_ products formation near the interfacial boundary of Cu‐based bimetallic catalysts. Conversely, the abundant Cu‐Ag interface may impede C_2+_ products formation, leading to a transformation in the catalytic selectivity from the C_2+_ products to CH_4_.

It has been reported that CO stripping voltammetry can serve as a robust electroanalytical technique for evaluating the CO binding energetics and surface coverage dynamics of catalysts [40, 41, 42, 43]. Thus, to elucidate the correlation between CO adsorption energetics and Cu‐Ag interfacial density, CO stripping experiments were performed. The anodic shift of stripping peaks serves as a descriptor for strengthened CO binding energy at the Cu‐Ag interface [44]. As illustrated in Figure 6b, the CO stripping curve of Cu‐Ag with an interface density of 10 cm^−2^ shows an oxidation peak emerging at 0.71 V vs. RHE, which is attributed to the oxidation reaction of adsorbed CO. Notably, as the Cu‐Ag interfacial density increased from 10 cm^−2^ to 100 cm^−2^, the CO oxidation peak shifted negatively by 0.02 V (from 0.71 V to 0.69 V), indicating a reduced binding strength of CO with a higher Cu‐Ag interface density. In other words, the Cu‐Ag interface lowers the CO binding strength and thus inhibits C─C coupling at a higher Cu‐Ag interface density, which is consistent with the trend observed in previous experiments.

### 2.5 Density Functional Theory Calculations

To better understand the effect of Cu–Ag interfaces on product formation (i.e., CH_4_ and C_2+_) in CO electroreduction, the constant‐potential density functional theory (DFT) calculations [45, 46] were performed. Herein, we mapped out two competing pathways: (i) ^*^CO hydrogenation to CH_4_ and (ii) ^*^CO dimerization to ^*^OCCO that leads to C_2+_ products. To faithfully capture the electrochemical environment at the metal–electrolyte interface and reveal the optimal reaction pathway for CORR [47, 48, 49], free energy diagrams were calculated on pure Cu and Cu‐Ag interfaces, respectively, under the identical potential (−1.6 V vs. SHE) as we used in the experimental work (Table S2).

**FIGURE 7:**
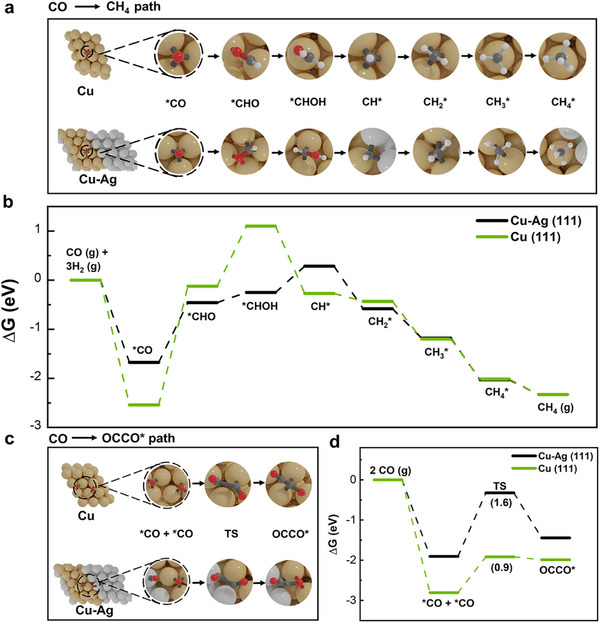
Theoretical calculations of electrocatalytic CORR on Cu (111) and Cu‐Ag (111) catalysts. (a) The processes of critical reaction intermediates in CO reduction to CH_4_;(b) Gibbs free energy diagrams for CO reduction to CH_4_; (c) The critical steps of ^*^CO dimerization to ^*^OCCO and (d) the corresponding Gibbs free energy diagrams. Color code: Cu, yellow, Ag, silver, H, white, C, black, O, red.

The major pathway in the CO‐to‐CH_4_ proceeds on both Cu surface and Cu–Ag interface via the following steps: ^*^CO → ^*^CHO → ^*^CHOH → ^*^CH→ ^*^CH_2_ → ^*^CH_3_ → CH_4_ (Figure 7a). Figure 7b shows that the initial hydrogenation of ^*^CO to ^*^CHO has the highest energy barrier in the pathway toward CH_4_, emerging as the most kinetically challenging step, whereas the subsequent hydrogenation steps of ^*^CH, ^*^CH_2_, and ^*^CH_3_ proceed without significant energetic barriers, which is in agreement with prior findings [50]. Notably, we found that the energy barrier of ∼1.2 eV was observed for this initial hydrogenation of ^*^CO to ^*^CHO on the Cu‐Ag boundary interface, which is significantly lower than that (∼2.4 eV) on the pure Cu surface. This low free energy barrier for the initial hydrogenation of ^*^CO indicates a more facile formation for CH_4_ on the Cu‐Ag surface in comparison with the pure Cu surface, which is consistent with the experimentally observed higher selectivity of CH_4_ with increasing Cu‐Ag interface densities.

Additionally, in the most accepted theory, the CO dimerization toward ^*^OCCO (Figure 7c) represents the key reaction step toward C_2+_ product formation [45, 46, 51, 52], and CO dimerization is the rate‐determining step for C─C bond formation. Thus, to simplify the computational scope regarding C_2+_ products, we focused exclusively on this elementary step and its associated transition state. As shown in Figure 7d, the pure Cu surface exhibits a lower reaction energy profile for the *CO dimerization step compared to the Cu‐Ag interface. The transition state analysis reveals that the activation barrier for ^*^CO dimerization on Cu is only 0.9 eV, whereas the corresponding barrier on Cu‐Ag rises significantly to 1.6 eV. These findings suggest that the ^*^CO dimerization is substantially hindered on the Cu‐Ag interface, leading to energetically unfavourable C─C bond formation on the interfacial sites, which agrees with the experimental finding that the formation of C_2+_ products was inhibited upon increasing Cu‐Ag interface densities.

Based on the above theoretical and experimental results, we therefore conclude that the Cu‐Ag boundary interface facilitates CH_4_ formation while inhibiting C_2+_ yields, which means that even if CO spills over to the adjacent Cu domains near the Cu‐Ag boundary interface, the Cu sites near the interface prefer to convert the migrated CO into CH_4_ instead of C_2+_. In other words, CO spillover through the Cu‐Ag interfacial boundary is unable to facilitate the C─C bond formation toward C_2+_ products.

We note that the present DFT calculations are based on static slab models and therefore do not explicitly capture dynamic surface reconstruction under reaction conditions. Solvation and electrochemical effects were incorporated using an implicit solvent model together with constant‐potential calculations (CP‐VASP combined with VASPsol), which provides a practical and widely adopted description of electrochemical interfaces. While explicit solvent models or ab initio molecular dynamics could further account for interfacial dynamics, their high computational cost places them beyond the scope of this work. Importantly, although such effects may influence absolute energetics, they are unlikely to alter the qualitative trends identified here, particularly for the increased barrier in ^*^CO─CO coupling and the relative promotion of CO hydrogenation at the Cu–Ag interface.

### 2.6 Implications of Unfavourable C─C Bond Formation from Binary Interface

In this work, we summarized the previous studies regarding the Cu‐based bimetallic catalysts, as shown in Table S3. It should be noted that although the majority of the Cu‐based bimetallic catalysts have stated that the CO spillover contributes to the enhanced C─C bond formation, all of these Cu‐based bimetallic catalysts were based on the nanostructured morphology (Table S3). In comparison with the Cu counterpart, the fabrication process for introducing CO‐selective metal catalysts into Cu may create complex reactive sites on the nanostructured binary catalysts, and the nanostructured morphology that relates to reactive sites may be changed when tuning the binary compositions, which may correlate with the improved C_2+_ formation. Additionally, most of the reported Cu‐based bimetallic catalysts were prepared via chemical methods [35, 53, 54, 55, 56, 57], thus, some residual chemicals on the binary catalysts may also play a part of role in the reaction pathways toward final products. [7, 36, 54]

If one would not have considered the above factors, it appears as if it was reasonable to directly link the enhanced C─C bond formation with CO spillover in the previous work. However, to obtain the Cu‐Ag binary catalysts with controllable binary interface densities, physical vapour deposition was employed in this work, which can rule out not only the morphology variation that relates to the creation of complex reactive sites but also residual chemicals. Through eliminating these factors, our results show that CO spillover through the binary interfacial boundary does not favour the C─C bond formation. Thus, the enhanced C_2+_ formation on bimetallic catalysts in previous work may not be mainly linked to the CO spillover effect, but to the creation of complex reactive sites on the nanostructured surface or even residual chemicals.

In addition to the possible creation of complex reactive sites on the nanostructured surface, it should be noted that the dynamic reconfiguration behaviour of Cu‐based bimetallic interface may also take place during CO_2_/CO reduction [36, 58, 59, 60, 61]. Particularly, the Chen group reported that Cu–Ag bimetallic nanowires undergo a dynamic reoxidation–reduction cycle involving Cu^+^ and Cu^0^, forming interfacial alloying and thus promoting CO_2_RR toward CH_4_ [62]. While we also found an increased CH_4_ formation with higher Cu‐Ag interface densities, CO spillover across the interface toward the adjacent Cu domain should not be significantly influenced by interfacial alloying based on the well‐known CO spillover hypothesis. Thereby, regardless of interface alloying, we still can conclude that CO spillover through the binary interfacial boundary is not a promoter for the C─C bond formation.

### 2.7 Conclusions and Perspectives

In this work, by a comparison between CO_2_ reduction and CO reduction on Cu‐Ag bimetallic catalysts with tunable binary interface densities, we demonstrated that CO spillover may not contribute to the C─C bond formation at the Cu‐Ag interface. Instead, the presence of the Cu‐Ag interface facilitates CH_4_ formation while suppressing C─C coupling. CO stripping experiments also suggest the inhibited C─C coupling at the Cu‐Ag interface. Additionally, our constant‐potential DFT calculations further corroborate that Cu‐Ag interfaces favor CO hydrogenation over CO dimerization, effectively steering the reaction pathway toward CH_4_ rather than C_2+_ products. Thereby, the improved formation of C_2+_ products via Cu‐based bimetallic catalysts in the previous work is unlikely to correlate with the CO spillover via the binary interface. This work not only provides new insights into the widely accepted CO spillover mechanism for CO_2_ electroreduction on Cu‐based bimetallic catalysts but also illustrates that isolating the effect of a single factor can help to get a better understanding of the underlying mechanism of catalysis.

## Author Contributions

M.M. conceived the original idea of this work. M.M. and Y.Y. supervised the manuscript. B.X. fabricated the catalysts and performed the experiments. X.D. and Y.W. performed HPLC to analyze liquid products. Z.L. performed the DFT calculations and analysis, and wrote the original computational sections of the manuscript. M.M. and B.X. wrote the original manuscript. All authors contributed to discussing the results and editing the manuscript. B.X. and Z.L. contributed equally to this work.

## Conflicts of Interest

The authors declare no conflicts of interest.

## Supporting information




**Supporting File**: advs74474‐sup‐0001‐SuppMat.docx.

## Data Availability

The data that support the findings of this study are available from the corresponding author upon reasonable request.
