# Supplementary material for: CO Spillover Is Not a Promoter for C─C Bond Formation in CO2 Electroreduction on Cu‐Ag Bimetallic Catalysts
Source: Adv Sci (Weinh). 2026 Mar 2;13(24):e20469. doi: 10.1002/advs.202520469 (PMC13116188; doi:10.1002/advs.202520469)
Supplement: Supplementary file 1 — Supporting File: advs74474‐sup‐0001‐SuppMat.docx. [file ADVS-13-e20469-s001.docx]

Supporting Information

### CO Spillover is Not a Promoter for C-C Bond Formation in CO_2_ Electroreduction on Cu-Ag Bimetallic Catalysts

Beining Xu,^1,#^ Zhaochun Liu,^2,#^ Xinjuan Du,^1^ Wen Yan^1^, Yunsong Yu,^1^*, Ionut Tranca,^2^ Frederik Tielens,^2^ and Ming Ma^1^*

^1^School of Chemical Engineering and Technology, Xi’an Jiaotong University, Xi’an 710049, People’s Republic of China

^2^General Chemistry (ALGC), Materials Modelling Group, Vrije Universiteit Brussel (VUB), Pleinlaan2, Elsene, 1050, Brussels, Belgium

^#^ These authors contributed equally to this work.

*Author to whom correspondence should be addressed.

E-mail address: [cloud.pine02@mail.xjtu.edu.cn](mailto:cloud.pine02@mail.xjtu.edu.cn); [mingma@xjtu.edu.cn](mailto:mingma@xjtu.edu.cn)

1. Experimental

1.1 Chemicals and Materials

Potassium bicarbonate (KHCO_3_, ≥99.99% metals basis) was purchased from Aladdin. Anion exchange membrane (AEM, Fumasep FAA-3-PK-75) and gas-diffusion layer (GDL, Sigracet 39 BB) were purchased from Fuel Cell Store. A nickel foam (Ni) anode and an Ag/AgCl counter electrode (saturated KCl) were employed in a flow cell (Gaoss Union CP10) were purchased from Gaoss Union Photoelectric technology Co., Ltd. The Cu target (99.99%) and Ag target (99.99%) for sputtering were purchased from Beijing ZhongNuo Advanced Material Technology Co., Ltd. All chemicals were used without further purification and ultrapure water (18.2 MΩ·cm, Master-S15UV) as a solvent for all experiments.

**1.2 Preparation and Characterization of Cu-Ag Bimetallic Catalysts**

1.2.1 Mask illustration

The deposition masks used in the deposition process were prepared through the utilization of a high-precision laser (minimum tolerance 0.003 mm) to get controllable slit widths of 0.1, 0.2, 0.5, 1 and 5 mm in Cu sheets (**Figure S1**), respectively. Additionally, to maintain the identical total surface area of sputtered Cu catalysts for different binary catalysts, the width of the slit is always equal to shield widths with varying widths (0.1, 0.2, 0.5, 1 and 5 mm) in the Cu sheets.


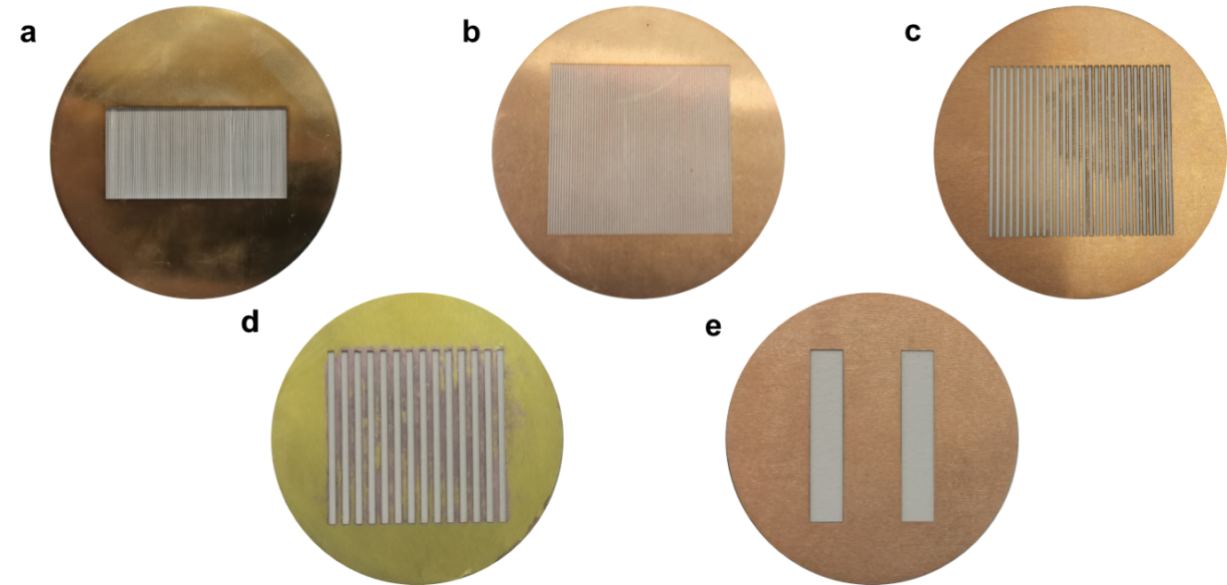


**Figure S1**. Photograph of the configuration of varying slit widths for the manufacture of deposition masks for Cu-Ag binary catalysts. The slit widths are as follows: (a) 0.1 mm, (b) 0.2 mm, (c) 0.5 mm, (d) 1 mm and (e) 5 mm.


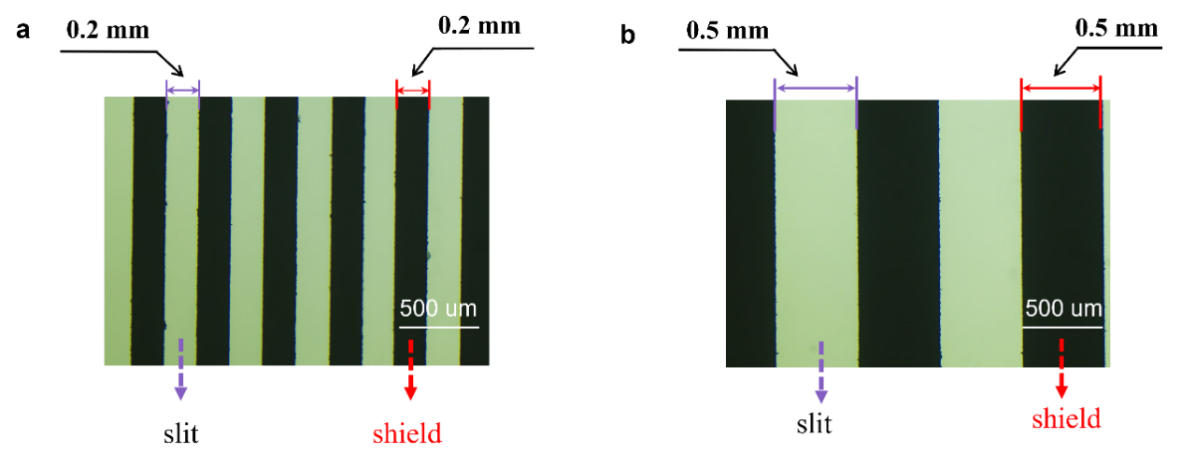


**Figure S2**. The Fluorescence Microscope photograph using a Nexcope (NIB610-FL) is representative of the slit width of deposition masks that are typically employed in the fabrication process for various interface density Cu-Ag binary catalysts. The widths of the slits and shields are identical for all masks, with the slit widths of (a) 0.2 mm and (b) 0.5 mm serving as illustrative examples.

Table S1. Correspondence between Cu-Ag bimetallic catalysts interface density and slit width of deposition mask.

| Slit width of the deposition mask (mm) | Interface density of Cu-Ag binary film (cm^-2^) |
| --- | --- |
| 0.1 | 100 |
| 0.2 | 50 |
| 0.5 | 20 |
| 1 | 10 |
| 5 | 2 |

**
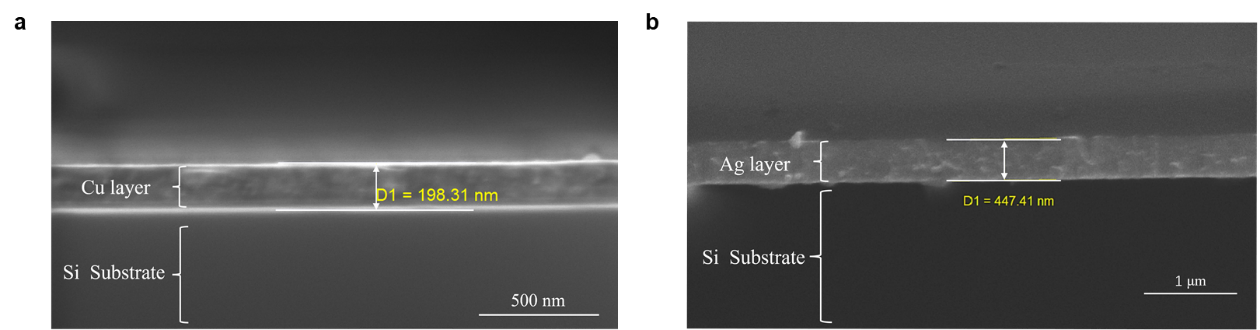
**

**Figure S3**. Cross-sectional SEM image of (a) Cu layer and (b) Ag layer deposited on Si.

In this work, the Cu/Ag catalyst layer was prepared via DC magnetron sputtering at 50 W with an argon pressure of 0.5 Pa under room temperature. To obtain the accurate deposition rate of Cu and Ag. Cu and Ag films were deposited on Si substrates, respectively. The thickness of the Cu/Ag layer can be controlled by the deposition time. Figure S3a, b shows the Cross-sectional SEM image of Cu/Ag on Si substrate deposited for 10 min, respectively, which indicates that ~200 nm thick Cu and ~450 nm thick Ag films were synthesized, corresponding to Cu and Ag deposition rates of ~20 nm/min and ~45 nm/min. Via controlling the deposition time, we synthesized Cu and Ag catalysts with the thickness of ~45 nm on GDEs.

**
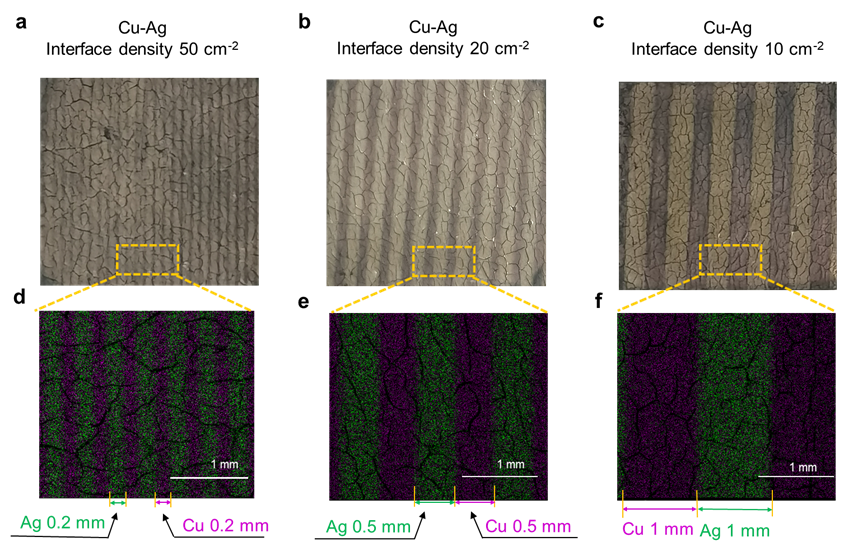
**

Figure S4. Characterization of Cu-Ag bimetallic catalysts after CO_2_ electrolysis. The digital photos and the related EDS elemental mapping images (Cu, purple, Ag, green) for the typical post CO_2_RR Cu-Ag binary samples with interface densities of (a, d) 50 cm^-2^, (b, e) 20 cm^-2^ and (c, f) 10 cm^-2^, respectively (The Cu and Ag catalysts were arranged alternately at equal intervals with 0.2 mm, 0.5 mm, and 1 mm, respectively).

After 90 minutes of CO_2_ reduction reaction (CO_2_RR) at 200 mA/cm^2^ in 1 M KHCO_3_, energy-dispersive x-ray spectroscopy (EDS) measurements were conducted using a TESCAN MAIA3 LMH operated at 15 keV were conducted. EDS mapping clearly revealed well-defined Cu-Ag interfaces (Fig. d-f) in representative Cu-Ag bimetallic catalysts with interfacial densities of 50 cm^-2^, 20 cm^-2^, and 10 cm^-2^ (Fig. a-c) after CO_2_ electrolysis. The Cu and Ag strips maintain the same alternate spacing. The widths of the strips are 0.2 mm, 0.5 mm and 1 mm, respectively. This demonstrates the stability and uniformity of the interfacial structure under electrochemical conditions.

1.3 Electrochemical Measurements

The CHI1140C potentiate was employed in all electrochemical tests with applied currents of -200 mA/cm² for 90 minutes. For all the electrochemical CO_2_/CO reduction were conducted in a flow cell, where the catholyte was separated from the anolyte by an Anion exchange membrane. The feed gas, comprising either CO_2_ or CO, was supplied continuously at a constant flow rate of 25 ml/min under the control of a digital mass flow controller.1 M KHCO_3_ was used as the electrolyte. An Ag/AgCl electrode and Ni foam were used, respectively, as the reference and the counter electrodes. Cu-Ag binary catalysts with a total surface area of 1 cm^2^² were employed as the working electrodes. During the electrolysis, the gas products mixed with unreacted CO_2_ or CO were directly vented into the gas-sampling loop of an online gas chromatograph (GC, PANNA A60) for periodic quantification. The liquid products dissolved in the given catholyte and anolyte reservoirs were collected and subsequently analyzed using high-performance liquid chromatography (HPLC, Agilent 1260).

1.4 Faradaic Efficiency of Gas Products

The gas products of CO_2_ reduction were analyzed using an online gas chromatograph. During electrolysis, the gas mixture from the outlet was directly channeled into the gas sampling loop of the GC for periodic quantification of gaseous products, with measurements taken at 10-minute intervals. The electrolysis process was consistently maintained at 90 minutes for all experiments, with gas analysis commencing at the 10th minute and repeated over seven cycles. The Faradaic efficiency (FE) for gaseous products was calculated using the following equation:

$$FE\left( \% \right)=\frac{Q_{gas}}{Q_{total}}\times100\%=\frac{n\times\phi\times C_{gas}\times F\times\frac{P}{RT}}{I}\times100\% (S1)$$

Where $Q_{gas}$ and $Q_{total}$ are charge transferred for gaseous product formation and charge passed through the working electrode, respectively. $n$ is the number of electrons required for producing one molecule of the related gas product. $\phi$ is the gas outlet flow rate from the gas chamber, $C_{gas}$ is the concentration of the gas product measured by gas chromatography, $F$ is the Faraday constant (96485 C/mol), $P$ is the ambient pressure, $R$ is the ideal gas constant, $T$ is the absolute temperature, and $I$ is the applied current. This approach ensured accurate and reproducible quantification of gaseous product yields.

1.5 Faradaic Efficiency of Liquid Products

After the completion of CO_2_/CO reduction, liquid products diluted in both catholyte and anolyte were collected and analyzed by HPLC. The Faraday efficiency of liquid product is calculated by the following equation:

$$FE\left( \% \right)=\frac{Q_{liquild}}{Q_{tatal}}\times100\%=\frac{n\times{F\times C}_{liquild\times}V_{liquild}}{I\times t}\times100\% (S2)$$

where $Q_{liquild}$ and $Q_{total}$ are charge transferred for liquid product formation and charge passed through the working electrode, respectively. $n$ is the number of electrons required for producing one molecule of the related liquid product. $F$ is the Faraday constant, $C_{liquild}$ is the concentration of the liquid product measured by HPLC, $V_{liquild}$ is the volume of electrolyte, $I$ is the applied current, and $t$ is the time of electrolysis. It should be noted that the electrolyte volume causes changes before and after the reaction, and this must be taken into account when calculating the liquid Faraday efficiency.^1^

1.6 CO_2_ Reduction Rate

Calculating the Faradaic efficiency for a single product can propagate variations from co-generated products. In this case, the error in Faradaic efficiency is primarily attributed to fluctuations in hydrogen measurement, which is the predominant byproduct of CO_2_RR. Although hydrogen does not consume CO_2_, it contributes to the total charge consumption, thereby affecting the accuracy of selectivity. Therefore, the CO_2_ consumption rate (mol cm^-2^ s^-1^) was employed as a more accurate metric for evaluating product selectivity, as it directly reflects the utilization of CO_2_ in the reaction. In the electrochemical conversion of CO_2_ can be reduced to various gaseous and liquid products, as shown in the reactions below.^2^

$$CO_{2}+H_{2}O+2e^{-}=CO+2OH^{-} (-0.11 V vs. RHE) (S3)$$

$$CO_{2}+{6H}_{2}O+8e^{-}=CH_{4}+8OH^{-} (0.17 V vs. RHE) (S4)$$

$$CO_{2}+H_{2}O+2e^{-}=HCOO^{-}+OH^{-} \left( -0.03 V vs. RHE \right) (S5)$$

$$2CO_{2}+{8H}_{2}O+12e^{-}=C_{2}H_{4}+12OH^{-} (0.08 V vs. RHE) (S6)$$

$$2CO_{2}+{5H}_{2}O+8e^{-}={CH}_{3}COO^{-}+7OH^{-} (-0.26 V vs. RHE) (S7)$$

$$2CO_{2}+{9H}_{2}O+12e^{-}=C_{2}H_{5}OH+12OH^{-} \left( 0.09 V vs. RHE \right) (S8)$$

Due to the above cathodic reactions (Equation S(3-8)), number of CO_2_ molecules required when 1 mole of a specific product (i) is generated can be expressed as:

$${CO_{2}}_{reduction rate}=\frac{{FE}_{i}\times Q_{total}\times m_{i}}{n_{i}\times F\times t}=\frac{{FE}_{i}\times I\times m_{i}}{n_{i}\times F} (S10)$$

where ${FE}_{i}$ is the Faradaic Efficiency for CO_2_-Derived Product i, $Q_{total}$ is charge passed through the cathode in 5400 s (i.e. 1080 C) $I$ is the applied current, $m_{i}$ is the number of CO_2_ molecules required to produce 1 mole of product i, $n_{i}$ is the number of electrons required for producing one molecule of the product i, $F$ is the Faraday constant and $t$ is the time of electrolysis.


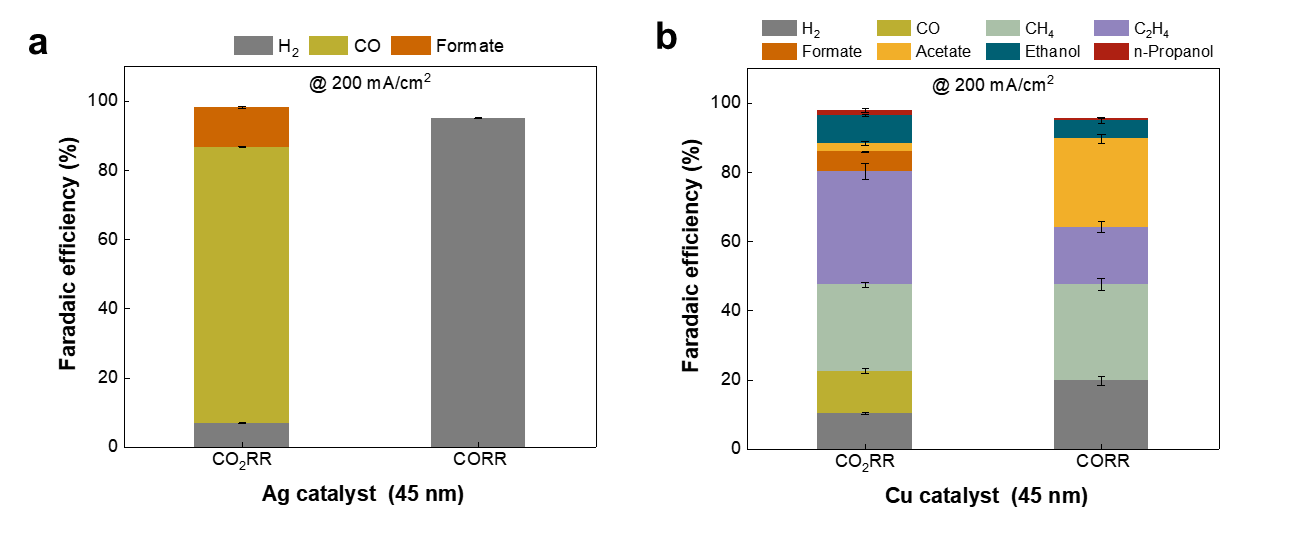


Figure S5. Electrochemical reduction of CO_2_ and CO performance on 45 nm (a) Ag and (b) Cu Catalysts in a flow cell with 1 M KHCO_3_ at 200 mA/cm^2^.

Ag has been demonstrated to be incapable of reducing CO_2_ to C_2+_ products. Instead, the primary product is CO, which exhibits a Faradaic efficiency (FE) of approximately 80% in the CO_2_RR. In the CORR, Ag does not participate in the reaction, and hydrogen is the sole product generated.

Compared to Cu-Ag bimetallic catalysts, it is true that pure Cu is more favorable for the generation of C_2+_ products.


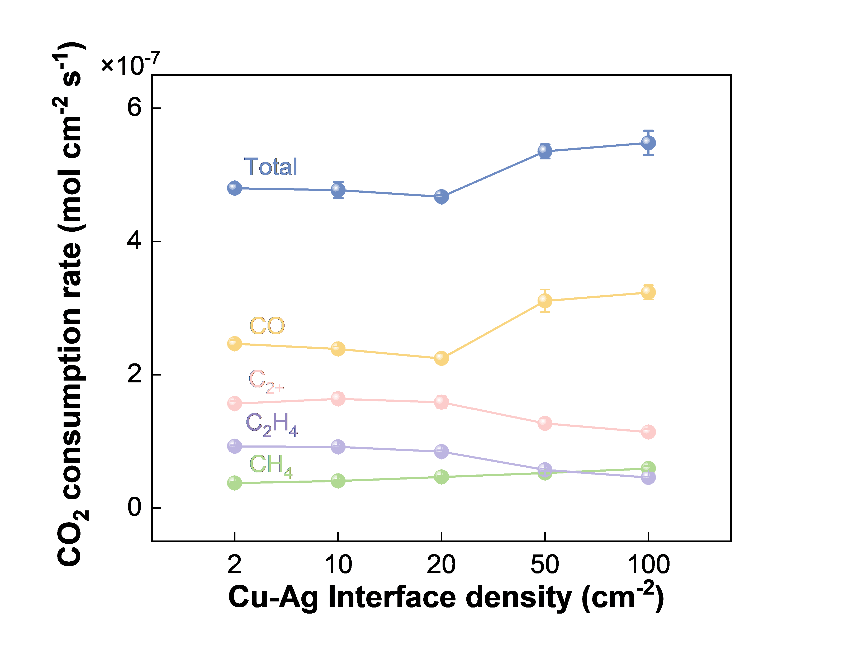


Figure S6. A comparison of the CO_2_ reduction rate towards CO, C_2_H_4_, CH_4_, C_2+_ products and CO_2_ reduction total products on Cu-Ag bimetallic catalysts with different interface density.

In this work, we also compared the CO_2_ reduction rate toward the products under different interface densities, as shown in **Figure S6**. While the CO_2_ consumption rate toward total C_2+_ steadily dropped when increasing interface densities, the total CO_2_ consumption rate for CO_2_ conversion gradually enhanced (total CO_2_ consumption rate was 5.35×10^-7^ mol cm^-2^ s^-1^). It appears that the total CO_2_ consumption for all products nearly follows the trend of CO_2_ reduction rate toward CO. While maximum CO FE was ~30% and total C_2_ FE was ~40%, CO_2_ consumption rate toward CO is much larger than that of total C_2+_ products, owing to that 1 CO_2_ molecule conversion into CO only requires 2 electrons. Thus, from the environmental perspective, electrochemical conversion of CO_2_ into CO should be a better avenue if the goal is to consume more greenhouse gas.

1.7 Electrochemical Impedance Spectroscopy Measurements

Potential electrochemical impedance spectroscopy was used on Cu-Ag bimetals catalysts (interface densities of 10 cm^-2^) in a GDE-type flow cell with 1 M KHCO_3_ aqueous solution to determine the solution resistance (Rs). During the experiment, the gas flow chamber was continuously fed with CO_2_ at a flow rate of 25 ml/min. The Impedance spectra were recorded using a constant potential instrument (Biologic) in the frequency range 100 kHz to 10 mHz with an amplitude of 10 mV at a fixed potential.


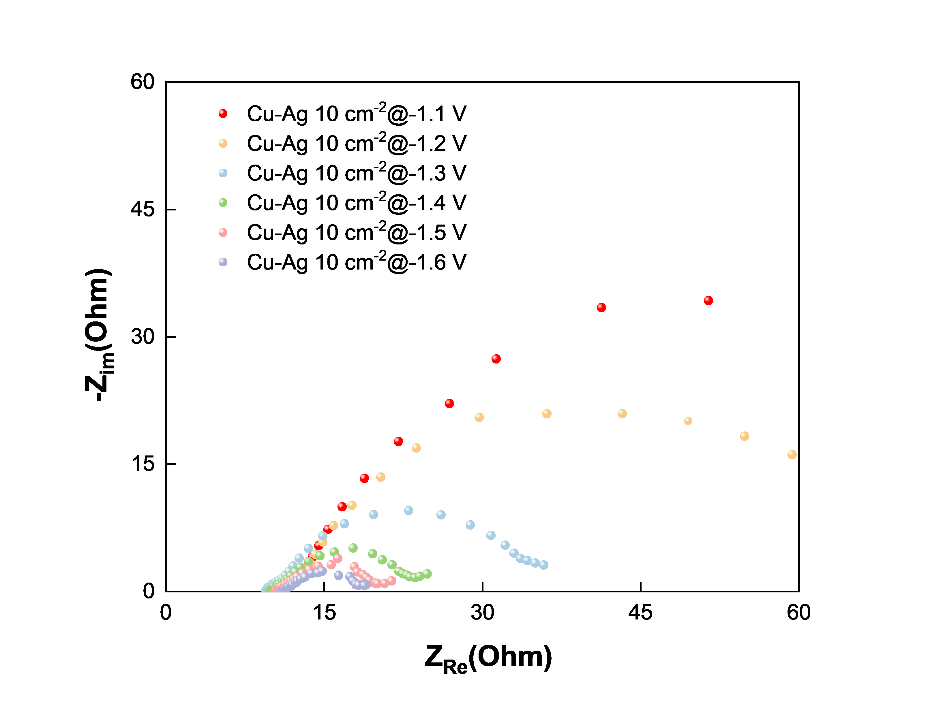


Figure S7. Nyquist plots of Cu-Ag bimetals catalysts (interface densities of 10 cm^-2^) in 1 M KHCO_3_ aqueous solution at various potentials.

The applied potentials vs the Ag/AgCl reference electrode were converted to the standard hydrogen electrode (SHE) reference scale with *i*R correction according to the following equation:

$$E_{(vs.SHE)}=E_{(vs.Ag/AgCl)}+0.197 V-i R_{S} (S11)$$

| Current (mA) | Rs (Ω) | Corrected V vs. SHE |
| --- | --- | --- |
| 200 | 10.543 | -1.594 |

Table S2 IR-corrected potentials in 1 M KHCO_3_.

1.8 CO Stripping Measurements

CO stripping was performed on Cu-Ag bimetallic catalysts with interface densities of 10 and 100 cm^-2^ in a GDE-type flow cell with 1 M KHCO_3_. Prior to the experiment, a pre-reduction treatment was carried out at -0.2 V vs RHE to eliminate oxides present on the surface of the Cu-Ag catalysts. Subsequently, pure CO gas was fed into the gas chamber for CO adsorption at a fixed potential of 0.1 V vs. RHE for five minutes. All the CV of CO stripping were collected after stopping CO gas at a scan rate of 20 mV s^-1^.

**2. DFT Calculations**

**2.1** **Computational Methods**

Spin-polarized Density Functional Theory (DFT) calculations were conducted using the Vienna Ab Initio Simulation Package (VASP 6.4.2) for this study.^3^ The ion-electron interactions were described employing the projector-augmented wave (PAW) method^4^ while the electron exchange-correlation was modeled using the Perdew-Burke-Ernzerhof (PBE) functional within the generalized gradient approximation (GGA).^5^ A plane-wave basis set with an energy cut-off of 400 eV was utilized. The convergence threshold for ionic steps in geometry optimization was set to 1 × 10^−5^ eV, and the optimization was considered converged when the forces on each atom were below 0.02 eV/Å. To validate that the optimized structures represented genuine minima, a frequency analysis was performed. Van der Waals interactions considered by employing the DFT-D3 method.^6^

Cu surfaces were modeled using periodic slab representations of the Cu (111) facet, constructed with a (4×3) surface supercell containing four atomic layers. The bottom two layers were fixed to mimic the bulk structure, while the top layers were allowed to relax. For the Cu–Ag interface, composite slabs were built by combining four-layer slabs of Cu (111) and Ag (111) surfaces, aligned within the same (4×3) supercell configuration. To minimize interfacial strain, the in-plane lattice parameters were fixed to those of the Ag (111) surface. During structural optimization, the bottommost layer of the interface was fixed, while all other atoms were fully relaxed. A vacuum region of 15 Å was introduced perpendicular to the surface to prevent spurious interactions between periodic images. Brillouin zone sampling was performed using a 5×3×1 Monkhorst–Pack mesh for Cu (111) slabs and a 1×1×1 mesh for the Cu–Ag interface, which was sufficient due to the large lateral supercell size. Constant-potential thermodynamic calculations related to the electrochemical CORR process were conducted using CP-VASP^7-8^ in combination with VASPsol.^9^

**2.2 DFT Calculations and Gibbs Free‐Energy Evaluation**

Under constant‐potential conditions, the Gibbs free‐energy change (ΔG) for each elementary step of the CORR was evaluated using a grand‐canonical framework. Specifically, we computed the Gibbs free energy change (∆G) at 298.15 K and 1 atm using

$$\Delta G=\Delta E\left( U \right)+\Delta E_{ZPE}+\Delta G_{U}^{PCET}+\Delta G_{pH}+\Delta G_{field}+\Delta\int_{0}^{298.15K} C_{P}dT-T\Delta S$$

$$(S12)$$

$\Delta E(U)$ represents a grand canonical energy of the system, while ∆E_ZPE_ denotes their zero-point vibrational energy, $\Delta G_{U}^{PCET}$ the free‐energy contribution from proton-coupled electron transfer (PCET) at applied potential U, $\Delta G_{pH}$ is the correction of the free energy associated with the proton concentration, such that $\Delta G_{pH}=2.303\times k_{B}T\times pH$, with the pH taken as zero here, and $\Delta G_{field}$ is contribution to the free energy of the electric field originating from the electrochemical double layer, which is typically neglected (with values around 0.015 eV). $C_{P}$ is the heat capacity at constant pressure, $\Delta S$ represents the entropy change at a given temperature T and the integration terms are computed using the vibrational energies of CORR intermediates. The entropies of gas-phase molecules in their free state were obtained from the NIST-JANAF thermochemical tables^10^.

The grand‐canonical electronic energy was computed as

$$E\left( U \right)=E_{DFT}+\Delta n^{CPS}\times\left( U-V_{sol}+\frac{\phi_{SHE}}{e} \right) (S13)$$

where E_DFT_ is the fixed‐charge total energy from DFT, $\Delta n^{CPS}$ is the number of electrons exchanged under constant potential, $V_{sol}$ the reference potential in the bulk electrolyte, and $\phi_{SHE}$ = −4.6 eV the work function of the standard hydrogen electrode.

**2.3 Potential‐Dependent (Constant‐Potential) Implementation**

Potential‐dependent simulations employed the grand canonical ensemble method of Duan and co‐workers^11^. In this approach, the total electron count n is treated as an additional degree of freedom alongside atomic coordinates $r$, and the system enthalpy is written as

$$H\left( r,n \right)=E\left( r,n \right)- \left( n-n_{0} \right)\phi(S14)$$

where $E\left( r,n \right)$ is the internal energy at fixed $\left( r,n \right)$, $n_{0}$ the total nuclear charge, and $\phi$ the effective counter‐electrode potential.

Using the standard hydrogen electrode ($E_{fS}$= −4.6 eV) as reference, the potential offset is defined by

$$\phi=-U+\frac{E_{fS}}{e} (S15)$$

with U the applied bias vs. SHE. As the work function of SHE measurement is scattered from 4.4 through 4.8 eV,^12^ the average value of 4.6 eV is taken for our calculations, which is also frequently used in several recent studies.^7,13^

The electron chemical potential then follows

$$\mu=\frac{\partial E}{\partial N}-\phi=\frac{E_{f}}{e}-\phi(S16)$$

Here, $E_{f}$ refers to the Fermi level of the working electrode with adsorbates. While most DFT packages can output $E_{f}$, it must be referenced to the vacuum level during post-processing. The full gradient of H $\left( r,n \right)$ is then given by ($\partial H$/$\partial r$, $\partial H$/ $\partial n$) = ($-f,\mu$), where $f$ is the atomic force vector and μ is the chemical potential.

Table S3 Summary of CO_2_RR performance of Cu-based bimetallic catalysts towards multi-carbon (C_2+_) products

| Cu-based catalysts  Composition | Catalyst type | FE(C_2+_) | Potential  [V_RHE_] | Current density (mA/cm^2^) | C_2+_/C_1_ | FE_(C2+)_  Cu-based/Cu or Cu_2_O | Ref |
| --- | --- | --- | --- | --- | --- | --- | --- |
| AgCu | single-atom alloy | 94 ± 4% | −0.65 | -720 | ~15 | 1.67 | ^14^ |
| Au/Cu | Au nanoparticles on a polycrystalline Cu foil | - | -0.75 | - | ~180 | 2 orders of magnitude  (CO_2_ reduction rates) | ^15^ |
| Au–Cu | Janus nanocrystals | 80.0% | - | -466.1 | ~7 | - | ^16^ |
| Cu_500_Ag_1000_ | physical mixture of Cu and Ag | - | -0.7 | -160 | - | 4 | ^17^ |
| Cu_3_Pd | phase-separated | 63% | -0.3 | -370 | - | - | ^18^ |
| CuAg | Nanoporous | 85% | −0.7 | ∼ − 300 | - | 1.6 | ^19^ |
| Cu/Ag | Cu nanosheets with a Cu (111) surface and Ag nanoparticles | 80% | −1.1 | ∼-600 | - | - | ^20^ |
| Cu−Ag | nanowires | ∼76% | −1.05 | -30 | - | 1.3 | ^21^ |
| Cu/Ag | nanoparticles | 87% | -0.78 | -287 | - | ~1.45 | ^22^ |
| Cu@Ag-2 | Core-Shell nanoparticles | 67.6% | −1.1 | -22.7 | - | 16.1 | ^23^ |
| Cu_98_Pd_2_ | phase-separated  bimetals with CuPd(111) interfaces | 75.6% | -1.15 | -200 | 10.76 | 1.32 | ^24^ |
| Ag_65_–Cu_35_ | Janus nanostructures with {100} facets | 72% | -1.2 | - | 4.8 | 1.5 | ^25^ |
| 5-Ag/Cu_2_O | Ag-Decorated Cu_2_O Nanocube | 65% | -0.98 | - | - | 1.2（Cu_2_O） | ^26^ |
| Ag_1_−Cu_1.1_ | Nanodimers | FE(C_2_H_4_) ~40% | −1.1 | - | - | 3.4 | ^27^ |
| Au-Cu | Janus nanostructures | ~67% | −0.75 | -290 | - | ~2.4 | ^28^ |
| CuAg _1:0.02_ | Janus nanostructure | 70% | -1.2 | -67.6 | 4.6 | 2.5 | ^29^ |

REFERENCES

(1) Zheng, Z.; Yao, Y.; Yan, W.; Bu, H.; Huang, J.; Ma, M. Mechanistic Insights into the Abrupt Change of Electrolyte in CO_2_ Electroreduction. *ACS Catal.* **2024**, *14* (8), 6328-6338.

(2) HORi.Y. *Electrochemical CO_2_ Reduction on Metal Electrodes*; Moder Aspects of Electrochemistry, Springer, 2008.

(3) Kresse, G.; Furthmüller, J.; Hafner, J. Theory of the crystal structures of selenium and tellurium: The effect of generalized-gradient corrections to the local-density approximation. *Physical Review B* **1994**, *50* (18), 13181-13185.

(4) Blöchl, P. E. Projector augmented-wave method. *Physical Review B* **1994**, *50* (24), 17953-17979.

(5) Perdew, J. P. B., K.; Ernzerhof, M. Generalized Gradient Approximation Made Simple. *Phys. Rev. Lett.* **1996**, *77*, 3865–3868.

(6) Grimme, S.; Antony, J.; Ehrlich, S.; Krieg, H. A consistent and accurateab initioparametrization of density functional dispersion correction (DFT-D) for the 94 elements H-Pu. *J. Chem. Phys.* **2010**, *132*, 154104.

(7) Zhao, X.; Liu, Y. Origin of Selective Production of Hydrogen Peroxide by Electrochemical Oxygen Reduction. *J. Am. Chem. Soc.* **2021**, *143* (25), 9423-9428.

(8) Yu, S.; Levell, Z.; Jiang, Z.; Zhao, X.; Liu, Y. What Is the Rate-Limiting Step of Oxygen Reduction Reaction on Fe–N–C Catalysts? *J. Am. Chem. Soc.* **2023**, *145* (46), 25352-25356.

(9) Mathew, K.; Kolluru, V. S. C.; Mula, S.; Steinmann, S. N.; Hennig, R. G. Implicit self-consistent electrolyte model in plane-wave density-functional theory. *J. Chem. Phys.* **2019**, *151*, 234101.

(10) Chase, M. W. *NIST-JANAF Thermochemical Tables*; American Chemical Society and American Institute of Physics Press, 1998.

(11) Duan, Z.; Xiao, P. Simulation of Potential-Dependent Activation Energies in Electrocatalysis: Mechanism of O–O Bond Formation on RuO_2_. *J. Phys. Chem. C* **2021**, *125* (28), 15243-15250.

(12) Trasatti, S. Structure of the metal/electrolyte solution interface: new data for theory. *Electrochim. Acta* **1991**, *36*, 1659-1667.

(13) Duan, Z.; Henkelman, G. Theoretical Resolution of the Exceptional Oxygen Reduction Activity of Au(100) in Alkaline Media. *ACS Catal.* **2019**, *9* (6), 5567-5573.

(14) Du, C.; Mills, J. P.; Yohannes, A. G.; Wei, W.; Wang, L.; Lu, S.; Lian, J.-X.; Wang, M.; Guo, T.; Wang, X.; et al. Cascade electrocatalysis via AgCu single-atom alloy and Ag nanoparticles in CO_2_ electroreduction toward multicarbon products. *Nat. Commun.* **2023**, *14*, 6142.

(15) Morales-Guio, C. G.; Cave, E. R.; Nitopi, S. A.; Feaster, J. T.; Wang, L.; Kuhl, K. P.; Jackson, A.; Johnson, N. C.; Abram, D. N.; Hatsukade, T.; et al. Improved CO_2_ reduction activity towards C_2+_ alcohols on a tandem gold on copper electrocatalyst. *Nat. Catal.* **2018**, *1* (10), 764-771.

(16) Zhang, T.; Zhang, B.; Zang, Y.; Zeng, P.; Li, Y.; Fan, H. J. A selectivity switch for CO_2_ electroreduction by continuously tuned semi-coherent interface. *Chem* **2024**, *10* (9), 2745-2760.

(17) Chen, C.; Li, Y.; Yu, S.; Louisia, S.; Jin, J.; Li, M.; Ross, M. B.; Yang, P. Cu-Ag Tandem Catalysts for High-Rate CO_2_ Electrolysis toward Multicarbons. *Joule* **2020**, *4* (8), 1688-1699.

(18) Ma, S.; Sadakiyo, M.; Heima, M.; Luo, R.; Haasch, R. T.; Gold, J. I.; Yamauchi, M.; Kenis, P. J. A. Electroreduction of Carbon Dioxide to Hydrocarbons Using Bimetallic Cu–Pd Catalysts with Different Mixing Patterns. *J. Am. Chem. Soc.* **2016**, *139* (1), 47-50.

(19) Hoang, T. T. H.; Verma, S.; Ma, S.; Fister, T. T.; Timoshenko, J.; Frenkel, A. I.; Kenis, P. J. A.; Gewirth, A. A. Nanoporous Copper–Silver Alloys by Additive-Controlled Electrodeposition for the Selective Electroreduction of CO_2_ to Ethylene and Ethanol. *J. Am. Chem. Soc.* **2018**, *140* (17), 5791-5797.

(20) Luan, P.; Dong, X.; Liu, L.; Xiao, J.; Zhang, P.; Zhang, J.; Chi, H.; Wang, Q.; Ding, C.; Li, R.; et al. Selective Electrosynthesis of Ethanol via Asymmetric C–C Coupling in Tandem CO_2_ Reduction. *ACS Catal.* **2024**, *14* (11), 8776-8785.

(21) Gao, J.; Zhang, H.; Guo, X.; Luo, J.; Zakeeruddin, S. M.; Ren, D.; Grätzel, M. Selective C–C Coupling in Carbon Dioxide Electroreduction via Efficient Spillover of Intermediates As Supported by Operando Raman Spectroscopy. *J. Am. Chem. Soc.* **2019**, *141* (47), 18704-18714.

(22) She, X.; Zhang, T.; Li, Z.; Li, H.; Xu, H.; Wu, J. Tandem Electrodes for Carbon Dioxide Reduction into C_2+_ Products at Simultaneously High Production Efficiency and Rate. *Cell Rep. Phys. Sci.* **2020**, *1* (4), 100051.

(23) Zhang, S.; Zhao, S.; Qu, D.; Liu, X.; Wu, Y.; Chen, Y.; Huang, W. Electrochemical Reduction of CO_2_ Toward C_2_ Valuables on Cu@Ag Core‐Shell Tandem Catalyst with Tunable Shell Thickness. *Small* **2021**, *17*, 2102293.

(24) Zhu, C.; Chen, A.; Mao, J.; Wu, G.; Li, S.; Dong, X.; Li, G.; Jiang, Z.; Song, Y.; Chen, W.; et al. Cu–Pd Bimetallic Gas Diffusion Electrodes for Electrochemical Reduction of CO_2_ to C_2+_ Products. *Small Struct.* **2023**, *4*, 2200328.

(25) Ma, Y.; Yu, J.; Sun, M.; Chen, B.; Zhou, X.; Ye, C.; Guan, Z.; Guo, W.; Wang, G.; Lu, S.; et al. Confined Growth of Silver–Copper Janus Nanostructures with {100} Facets for Highly Selective Tandem Electrocatalytic Carbon Dioxide Reduction. *Adv. Mater.* **2022**, *34*, 2110607.

(26) Herzog, A.; Bergmann, A.; Jeon, H. S.; Timoshenko, J.; Kühl, S.; Rettenmaier, C.; Lopez Luna, M.; Haase, F. T.; Roldan Cuenya, B. Operando Investigation of Ag‐Decorated Cu_2_O Nanocube Catalysts with Enhanced CO_2_ Electroreduction toward Liquid Products. *Angew. Chem. Int. Ed.* **2021**, *60* (13), 7426-7435.

(27) Huang, J.; Mensi, M.; Oveisi, E.; Mantella, V.; Buonsanti, R. Structural Sensitivities in Bimetallic Catalysts for Electrochemical CO_2_ Reduction Revealed by Ag–Cu Nanodimers. *J. Am. Chem. Soc.* **2019**, *141* (6), 2490-2499.

(28) Zheng, Y.; Zhang, J.; Ma, Z.; Zhang, G.; Zhang, H.; Fu, X.; Ma, Y.; Liu, F.; Liu, M.; Huang, H. Seeded Growth of Gold–Copper Janus Nanostructures as a Tandem Catalyst for Efficient Electroreduction of CO_2_ to C_2+_ Products. *Small* **2022**, *18*, 2201695.

(29) Zhang, S.; Zhang, B.; Yang, S.; Shao, T.; Li, X.; Cao, R.; Cao, M. Nanoscale Cu–Ag Heterostructures for CO_2_ Reduction to C_2+_ Products. *ACS Appl. Nano Mater.* **2025**, *8* (4), 1893-1902.
